# Surveillance of respiratory viruses in the outpatient setting in rural coastal Kenya: baseline epidemiological observations

**DOI:** 10.12688/wellcomeopenres.14662.1

**Published:** 2018-07-25

**Authors:** Joyce Uchi Nyiro, Patrick Munywoki, Everlyn Kamau, Charles Agoti, Alex Gichuki, Timothy Etyang, Grieven Otieno, D. James Nokes

**Affiliations:** 1 Virus Epidemiology and Control, KEMRI-Wellcome Trust Research programme, Kilifi, +254, Kenya; 2 Public Health, Pwani University, Kilifi, +254, Kenya; 3 School of Life Sciences and Zeeman Institue of Systems Biology and Infectious Disease Research (SBIDER), University of Warwick, Conventry, UK

**Keywords:** Outpatient; Respiratory viruses; Surveillance; Real-time PCR; Nasopharyngeal samples; Coastal Kenya

## Abstract

**Background: **Endemic and seasonally recurring respiratory viruses are a major cause of disease and death globally. The burden is particularly severe in developing countries. Improved understanding of the source of infection, pathways of spread and persistence in communities would be of benefit in devising intervention strategies.

**Methods: **We report epidemiological data obtained through surveillance of respiratory viruses at nine outpatient health facilities within the Kilifi Health and Demographic Surveillance System, Kilifi County, coastal Kenya, between January and December 2016. Nasopharyngeal swabs were collected from individuals of all ages presenting with acute respiratory infection (ARI) symptoms (up to 15 swabs per week per facility) and screened for 15 respiratory viruses using real-time PCR. Paediatric inpatient surveillance at Kilifi County Hospital for respiratory viruses provided comparative data.

**Results:** Over the year, 5,647 participants were sampled, of which 3,029 (53.7%) were aged <5 years. At least one target respiratory virus was detected in 2,380 (42.2%) of the samples; the most common being rhinovirus 18.6% (1,050), influenza virus 6.9% (390), coronavirus 6.8% (387), parainfluenza virus 6.6% (371), respiratory syncytial virus (RSV) 3.9% (219) and adenovirus 2.7% (155). Virus detections were higher among <5-year-olds compared to older children and adults (50.3% vs 32.7%, respectively; χ ^2^(1) =177.3, P=0.0001). Frequency of viruses did not differ significantly by facility (χ ^2^(8) =13.38, P=0.072). However, prevalence was significantly higher among inpatients than outpatients in <5-year-olds for RSV (22.1% vs 6.0%; χ ^2^(1) = 159.4, P=0.0001), and adenovirus (12.4% vs 4.4%, χ ^2^(1) =56.6, P=0.0001).

**Conclusions:** Respiratory virus infections are common amongst ARI outpatients in this coastal Kenya setting, particularly in young children. Rhinovirus predominance warrants further studies on the health and socio-economic implications. RSV and adenovirus were more commonly associated with severe disease. Further analysis will explore epidemiological transmission patterns with the addition of virus sequence data.

## Introduction

Acute respiratory infection (ARI) is a major cause of morbidity and mortality globally, principally affecting young children and the elderly, and with majority of the burden occurring in low-resource settings ^1, 2^. Viruses are recognized as major cause of both mild ARI and of severe acute lower respiratory tract infection (LRTI) ^3^. Respiratory syncytial virus (RSV), rhinovirus and influenza A are often identified as the most common viruses associated with ARI ^4, 5^, but a wide range of viruses are to be found in ARI presentations to the hospital and outpatient settings ^5^. The changing landscape of ARI due to the widespread use of conjugated bacterial vaccines could lead to an increased prominence of viral causes of these illnesses ^6, 7^.

Given this context, greater emphasis on the control of virus-associated ARI is likely. Vaccination as an intervention for the control of viral respiratory infections faces considerable hurdles. These include continuous or rapid pathogen evolution (e.g. influenza) ^8, 9^, high serotype diversity (e.g. rhinovirus) ^10^ or target populations not appropriate for current vaccines (e.g. RSV) ^11^. Consequently, innovation in vaccination strategies for these pathogens (e.g. targeting of schools and households), mass use of antivirals or non-pharmacological methods such as social distancing (e.g. school closures), are options to consider. Designing strategies to control respiratory viruses would be assisted by detailed knowledge of the patterns of spread, i.e. of distinct pathways of transmission in communities, at various social organizational levels from the household, school, local community, nationally and beyond country boundaries.

The present work forms part of a larger project under the title SPReD (Studies of the Pathways of transmission of Respiratory virus Disease), which aims to map patterns of spread of a range of respiratory viruses using epidemiological and nucleotide sequence data across different settings in Kenya. Here we present the baseline results of 1 year of respiratory virus epidemiological surveillance at health facilities within a well-defined coastal Kenyan population and a comparison with contemporaneous data on inpatient admissions to Kilifi County Hospital (KCH).

## Methods

### Study site

This study was conducted on the coast of Kenya, within the Kilifi Health and Demographic Surveillance System (KHDSS) ^12– 14^. The KHDSS area was defined and mapped for demographic surveillance, clinical and epidemiological research by the KEMRI Wellcome Trust Research Programme (KWTRP) in the year 2000. It is located in Kilifi County along the coastal fringe and covers an area of 891 km ^2^, 50 km north and south, and 30 km west, of the KCH. The KHDSS monitors a population of around 296,000 residents (2016 census) through household enumeration visits conducted every 4 months. The major economic activity of most residents is subsistence farming of maize, cassava, cashew nuts and coconuts, as well as goats and dairy cattle ^14^.

**Figure 1.**
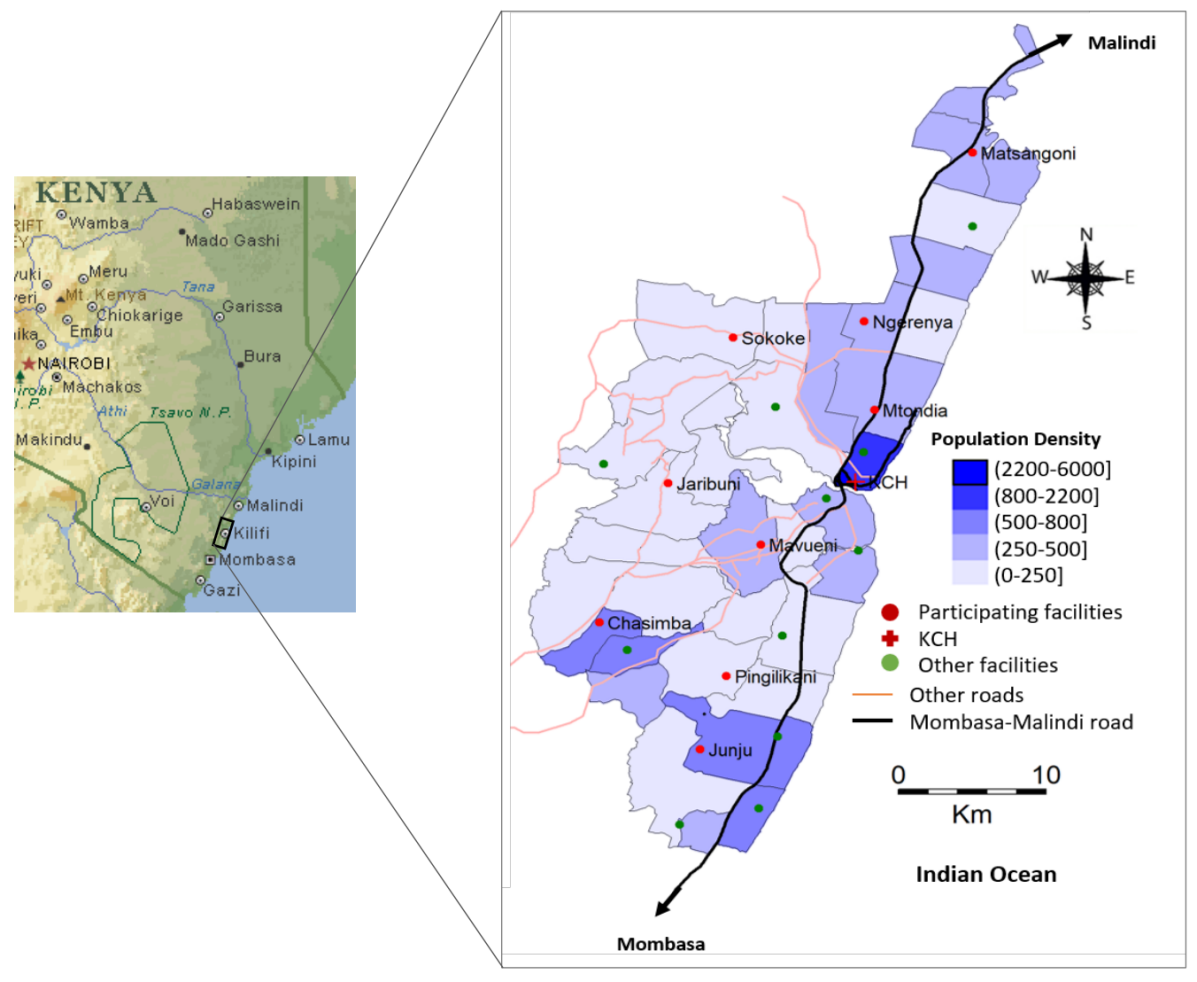
A map of the Kilifi Health and Demographic Surveillance System (HDSS) area, coastal Kenya, expanded from a map of Kenya, showing population density (person per Km ^2^) and the health facilities where the study was conducted in 2016. The red circles show the nine participating health facilities while the green markers show the other public health facilities within the KHDSS area.

The KHDSS area has 21 public health facilities (including the KCH) receiving out-patients, which operate under the Kenya Ministry of Health (MoH). In total, nine of these facilities were selected for this study: Matsangoni, Ngerenya, Mtondia, Sokoke, Mavueni, Jaribuni, Chasimba, Pingilikani and Junju ( Figure 1). The facilities were purposively selected to provide a broad representation across the geographical region, covering major road networks into the location and variation in population density. All specimen processing and testing was carried out at KEMRI-Wellcome Trust Research Programme laboratories in Kilifi.

### Patient recruitment and specimen collection

Participant recruitment and specimen collection was integrated within the routine patient care at the nine selected outpatient facilities led by a resident clinician or nurse. Each facility had one or two sampling days per week, usually scheduled from Monday to Friday, between 9.00 am and 1.00 pm. On each sampling day, a study fieldworker stationed at the health facility, assisted by the resident clinician or nurse, would describe the study to the attending patients. Any person present with listed signs and symptoms of ARI would be asked to see the fieldworker for further screening and obtainment of consent as they await review by the nurse or clinician. Patients of any age presenting with one or more ARI symptoms of cough, sneezing, nasal congestion, difficulty breathing, or increased respiratory rate for age (as defined by the World Health Organisation ^15^) were eligible. New-borns aged less than 7 days and patients with ARI for more than 30 days were excluded. Written individual informed consent was sought from adult patients and parents/guardians of patients below 18 years. The study commenced in December 2015 with piloting before enhanced surveillance of 15 samples per site per week from January to December 2016.

The sample size of 15 samples per site per week was determined based on outpatient data on the number of respiratory infection cases seen per month per selected health facility and our previous experience with inpatient surveillance for respiratory viruses at KCH. To optimize the study power to detect a diversity of the respiratory viruses, we estimated to collect approximately 7000 NPS specimen within a 1-year study period. Out of the 7000 NPS, we expected 1050 (15%), 700 (10%), 490 (7%) and 490 (7%) specimens to be positive for rhinoviruses, RSV, coronaviruses, and influenza viruses, respectively.

The selection of participants each week was on a ‘first-come first-served’ basis as they presented to the health facility on the set sampling days. A standardised questionnaire ( Supplementary File 1) was used to collect biodata, presenting symptoms as well as the treatment provided which was entered directly into a study database using a computer tablet. A nasopharyngeal swab (NPS) was collected from each participant by inserting a sterile nylon-flocked plastic-shafted swab (503CS01, Copan Diagnostics, Flocked Swab Technologies, Italy) into one nostril to a distance where the tip located in the deep nasopharynx and was twisted 3 times before it was gently removed (in total taking about 10 seconds) ^16^. The NPS sample was stored in universal virus transport media (Copan Diagnostics, USA) and kept at approximately 8°C in an ice-packed cool box for return to KWTRP within 4 h of collection.

### Ethical considerations

All individuals, parents and guardians gave written informed consent for themselves or their children to participate in this study. The study was approved by the KEMRI-Scientific and Ethical Review Unit (SERU# 3103) and the University of Warwick Biomedical and Scientific Research Ethics Committee (BSREC# REGO-2015-6102).

### Laboratory procedures

NPS collections received at KWTRP virology laboratory were stored in 2-ml vials at −80°C until use. Using previously described methods ^17– 19^, RNA was extracted from the respiratory specimens by Qiacube HT using an RNeasy extraction kit (Qiagen, Germany) and screened for RSV (A and B), rhinovirus (HRV), human coronaviruses (OC43, NL63, E229), influenza viruses (FLU-A, B, and C), parainfluenza viruses (PIV 1-4), adenovirus (ADV) and human metapneumovirus (HMPV), using a multiplex real-time PCR assay system. Samples with cycle threshold (Ct) of <35.0 were defined as positive for the target virus. Residual NPS samples were stored at −80°C.

### Statistical analysis

Statistical analysis was conducted using STATA version 13.1 (College Station, Texas). Summary statistics were produced for the data to give the proportions of virus positives by age and by location. Comparative data was obtained from the paediatric ward of KCH for patients aged <5 years admitted with acute LRTI from a contemporaneous respiratory virus surveillance ^20– 23^. Chi-squared and Fisher’s exact tests were used to test associations of virus occurrence with age, calendar month, facility, setting (outpatient or inpatient) and other demographic characteristics. Frequency distribution graphs were generated for all virus targets. Graphs for temporal patterns for each virus were generated.

## Results

### Baseline characteristics of participants and sites

A total of 5647 participants were recruited from January to December 2016. The median age was 4 years (interquartile range (IQR): 1–15 years), 53.6% (n=3029) were children under 5 years of age, and 42.3% (2389) were male. The frequency of distribution of symptoms among participants by site, age and virus target are shown in Table 1. Although the proportions of each symptom by health facility, age category and virus target, were often statistically significantly different ( Table 1), the rank order of magnitude of each sign or symptom was approximately the same. A majority of participants (85.3%, n=4819), presented with symptoms of cough and nasal discharge. A history of fever was identified in n=3541 participants (62.7%), but declined with age from 10–19 years onwards. Symptoms indicative of lower respiratory tract involvement, crackles, chest indrawing and fast breathing for age were uncommon (1.2%, n=65). The number of participants in each facility for this study differed significantly by the month in which recruitment occurred (χ ^2^(11) =78.26, P=0.001). There were interruptions during the 5 ^th^ to 13 ^th^ of December 2016 due to a health workers’ industrial dispute and 1 week during the Christmas break that led to lower recruitment for that month. Only 56 participants were recruited in December from all the nine facilities out of the 585 expected per month.

**Table 1.** Distribution of respiratory symptoms among study participants from a study of 9 outpatient health facilities in Kilifi County coastal Kenya over the year 2016.

Characteristic	Fever, %	Chest indrawing, %	Crackles, %	Wheeze, %	Cough, %	Nasal discharge, %	Nasal flare, %	Difficulty breathing, %	Total participants, n
**Health Facility**									
Matsangoni	58.4	0.91	0.45	1.82	93.8	68.68	3.48	7.87	**661**
Ngerenya	58.1	0.48	0.8	1.6	96.3	81.28	0.8	11.36	**625**
Sokoke	51.5	0.81	1.3	1.63	97.1	80.33	1.46	8.62	**616**
Mtondia	68.8	1.17	0.58	0.73	96.1	63.41	0.87	5.1	**686**
Mavueni	68.5	0.78	0.78	1.1	94.2	81.38	5.16	7.82	**639**
Jaribuni	57.8	0.67	0.5	2.68	97.3	82.58	0.67	6.03	**597**
Chasimba	53.7	0.97	0.97	1.46	97.1	72.65	0.16	6.8	**618**
Junju	64.7	0.33	2.12	3.59	90.2	78.92	0.82	11.27	**612**
Pingilikani	83	0.84	3.04	3.37	92.6	72.18	1.35	21.25	**593**
**χ ^2^ P value**	0.0001	0.835	0.0001	0.001	0.0001	0.0001	0.0001	0.0001	
**Age Category**									
0–5 mths	63.48	1.91	1.72	2.29	91.97	78.78	4.21	12.62	**523**
6–11 mths	69.69	0.58	1.16	0.97	95.16	80.66	3.09	10.64	**517**
12–23 mths	74.64	1.43	2.21	3.64	96.1	82.31	2.6	12.35	**769**
24–35 mths	73.91	1.27	0.91	3.99	95.47	80.98	2.36	7.79	**552**
3–4 yrs	75.52	0.6	0.9	1.95	96.26	77.54	1.35	9.13	**668**
5–9 yrs	71.63	0.26	1.04	0.78	95.21	72.15	0.39	6.61	**772**
10–19 yrs	55.22	0.43	0.71	1.28	93.87	71.75	1	6.99	**701**
20–49 yrs	39.87	0.38	0.89	1.77	94.43	68.35	0.38	9.87	**791**
50–100 yrs	30.51	0.28	0.56	0.56	96.33	66.95	0.28	10.17	**354**
**χ ^2^ P value**	0.0001	0.005	0.127	0.0001	0.019	0.0001	0.0001	0.0001	
**Virus Target**									
RSV	85.39	2.74	1.37	3.65	95.89	72.6	3.2	13.7	**219**
Rhino	60.35	1.16	1.26	2.61	94.97	79.79	2.03	10.54	**1034**
Adeno	82	1	1	0	93	68	1	8	**100**
Flu	77.9	0	0.85	2.55	97.17	78.47	0.28	7.37	**353**
Corona	64.62	0	0.36	0.72	96.39	80.14	0.36	5.42	**277**
PIV	71.74	3	2.48	3.11	98.45	81.68	3.11	13.04	**322**
HMPV	70.67	0	1.33	0	100	77.33	1.33	6.67	**75**
**χ ^2^ P value**	0.0001	0.02	0.398	0.117	0.02	0.024	0.026	0.007	
**Sex**									
Male	68.63	1.05	1.67	2.01	95.53	77.98	1.92	10.12	**2389**
Female	58.36	0.58	0.77	1.93	95.56	73.72	1.44	8.94	**3258**
**χ ^2^ P value**	0.0001	0.051	0.002	0.841	0.105	0.0001	0.159	0.129	

### Virus detection

The prevalence of NPS collections positive for one or more respiratory virus target was 42.1% (n=2380). The median age of the virus-positive patients was 2 years (IQR, 1–9). The proportion of virus-positive individuals differed significantly by age, sex and education status, but not by sampling site ( Table 2). The prevalence of respiratory virus detections was higher among young children (<5 years) than older children and adults (≥5 years) (50.3% vs 32.7%, respectively; χ ^2^ (1) =177.3, P=0.0001). Infants aged below 6 months with ARI constituted 9.3% (n=523) of the participants, and 58% (302) of these had one or more respiratory virus targets detected. Of all virus-positive individuals, 70% (1665/2380) were not in any form of education, with the vast majority of these pre-school, i.e. 83.2% (1385/1665). During study piloting (month of December 2015), respiratory samples from 153 participants were collected; 24.2% (n=37) were children under 5 years of age and 37.9% (n=58) were positive for one or more virus.

**Table 2.** Characteristics of respiratory virus positive and negative participants from a study of 9 outpatient health facilities in Kilifi County coastal Kenya over the year 2016.

Characteristic	NPS virus positive(n)	%	NPS virus negative (n)	%	Total(n)	P value
	(n=2380)	42.2	(n=3267)	57.8	(n=5647)	
**Age in years**						
Mean	8.2		14.3		11.7	
Median (IQR*)	2 (1-9)		5 (1-20)		4 (1-15)	
**Sex**						
Male	1075	45	1314	55	2389	0.0001
Female	1305	40.1	1953	59.9	3258	
**Age Category**						
0–5 mths	302	57.74	221	42.26	523	0.0001
6–11 mths	276	53.38	241	46.62	517	
12–23 mths	398	51.76	371	48.24	769	
24–35 mths	276	50	276	50	552	
3–4 yrs	271	40.57	397	59.43	668	
5–9 yrs	303	39.25	469	60.75	772	
10–19 yrs	242	34.52	459	65.48	701	
20–49 yrs	234	29.58	557	70.42	791	
50–100 yrs	78	22.03	276	77.97	354	
**Education**						
Not in school	1665	45.91	1962	54.09	3627	0.001
Kindergaten	244	35.78	438	64.22	682	
Primary	412	35.61	745	64.39	1157	
Secondary	54	33.33	108	66.67	162	
Tertiary	5	26.32	14	73.68	19	
**Health Facility**						
Matsangoni	282	42.66	379	57.34	661	0.072
Ngerenya	263	42.08	362	57.92	625	
Sokoke	255	41.4	361	58.6	616	
Mtondia	317	46.21	369	53.79	686	
Mavueni	293	45.85	346	54.15	639	
Jaribuni	237	39.7	360	60.3	597	
Chasimba	259	41.91	359	58.09	618	
Junju	247	40.36	365	59.64	612	
Pingilikani	227	38.28	366	61.72	593	

**IQR**, Interquartile range; Chi2 test P value

**Figure 2.**
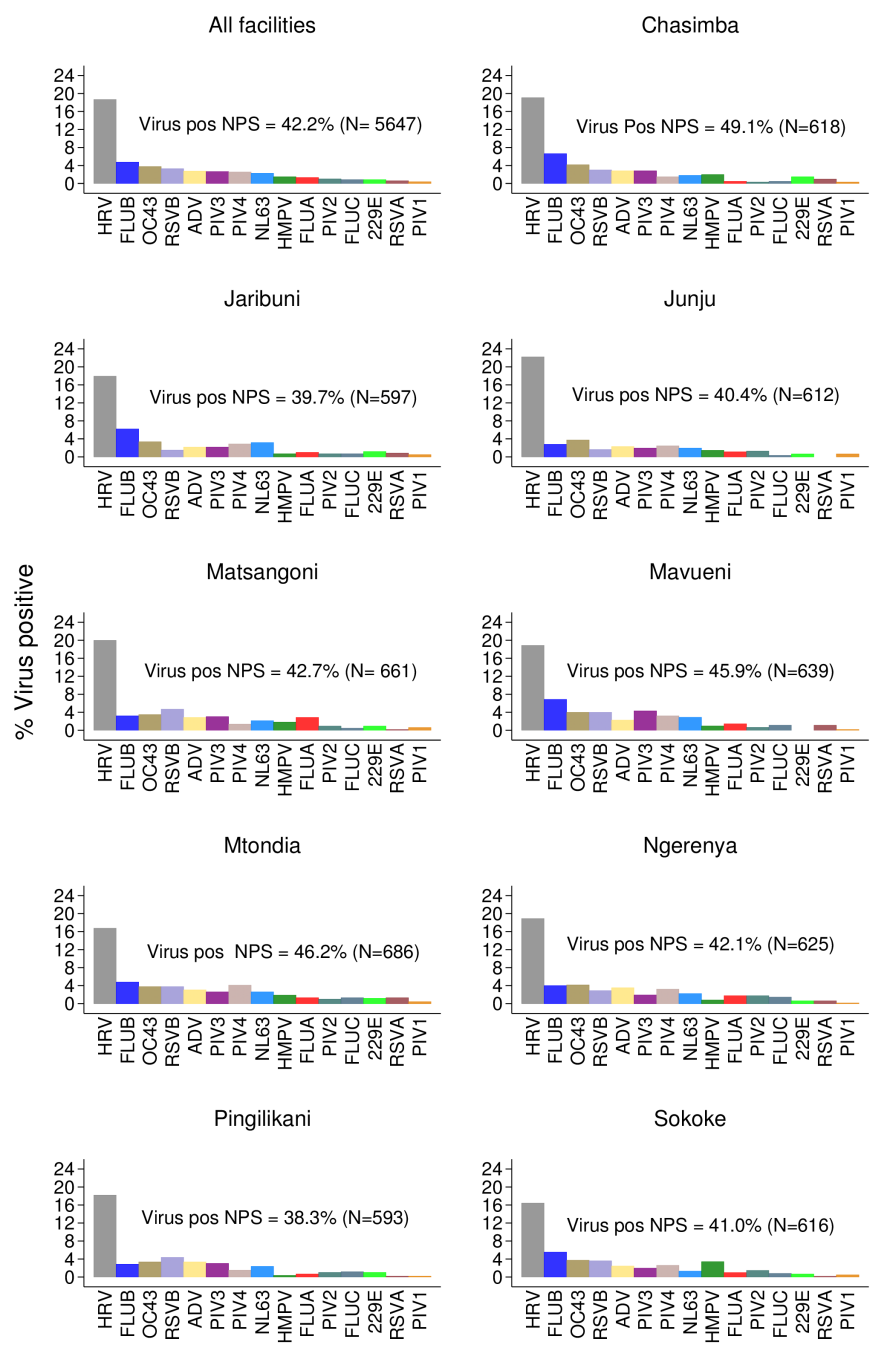
Percentage of nasopharyngeal swab samples positive for each of 15 respiratory virus targets for the nine health facilities (all facilities together and each individually) from ARI surveillance in the Kilifi Health and Demographic Surveillance System, Kenya, January to December 2016. RSVA, RSV group A; RSVB. RSV group B; HRV, human rhinovirus; PIV1-PIV4, parainfluenza virus types 1–4; ADV, adenovirus; OC43, human coronavirus OC43; NL63, human coronavirus NL63; E229, human coronavirus E229; FLUA-FLUC, influenza viruses types A-C; HMPV, human metapneumovirus.

**Figure 3.**
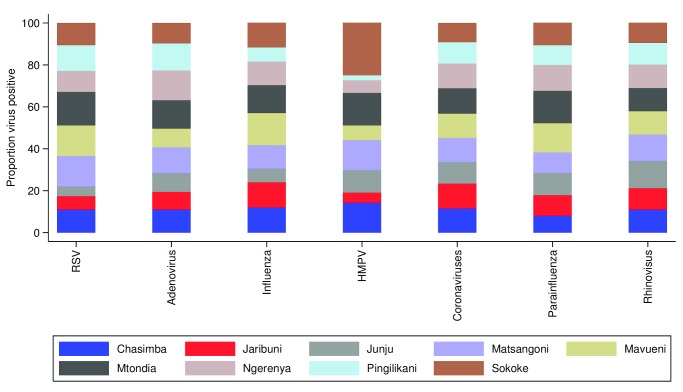
The relative proportion of nasopharyngeal swab samples positive across nine health facilities, by virus group, through surveillance of ARI presentations within the Kilifi Health and Demographic Surveillance System, Kenya, January to December 2016. RSV, RSV A and B groups; Influenza, influenza A, B and C types; Coronaviruses, coronavirus OC43, NL63, 229e; Parainfluenza, parainfluenza types 1–4.

The most common respiratory viruses detected in this study were rhinovirus 18.6% (n=1050), influenza virus 6.9% (n=390), coronavirus 6.8% (n=387), parainfluenza virus 6.6% (n=371), RSV 3.9% (n=219) and adenovirus 2.7% (n=155). The frequency of viruses did not differ significantly by facility of recruitment (χ ^2^(8) = 13.38, P=0.072) ( Table 2). The distribution of proportions of viruses detected from each health facility were similar, with rhinovirus being the most common virus detected from all the sites ( Figure 2). Furthermore, for each virus group, the proportion of all positive individuals were distributed roughly evenly across all health facilities, with the exception of HMPV ( Figure 3). Note for comparison that during the pilot phase of December 2015 during which 56 samples were collected, influenza virus type A; 37.9% (n=22), RSV; 20.6% (n=12) and rhinovirus; 17.2% (n=10) were the most commonly detected viruses.

**Figure 4.**
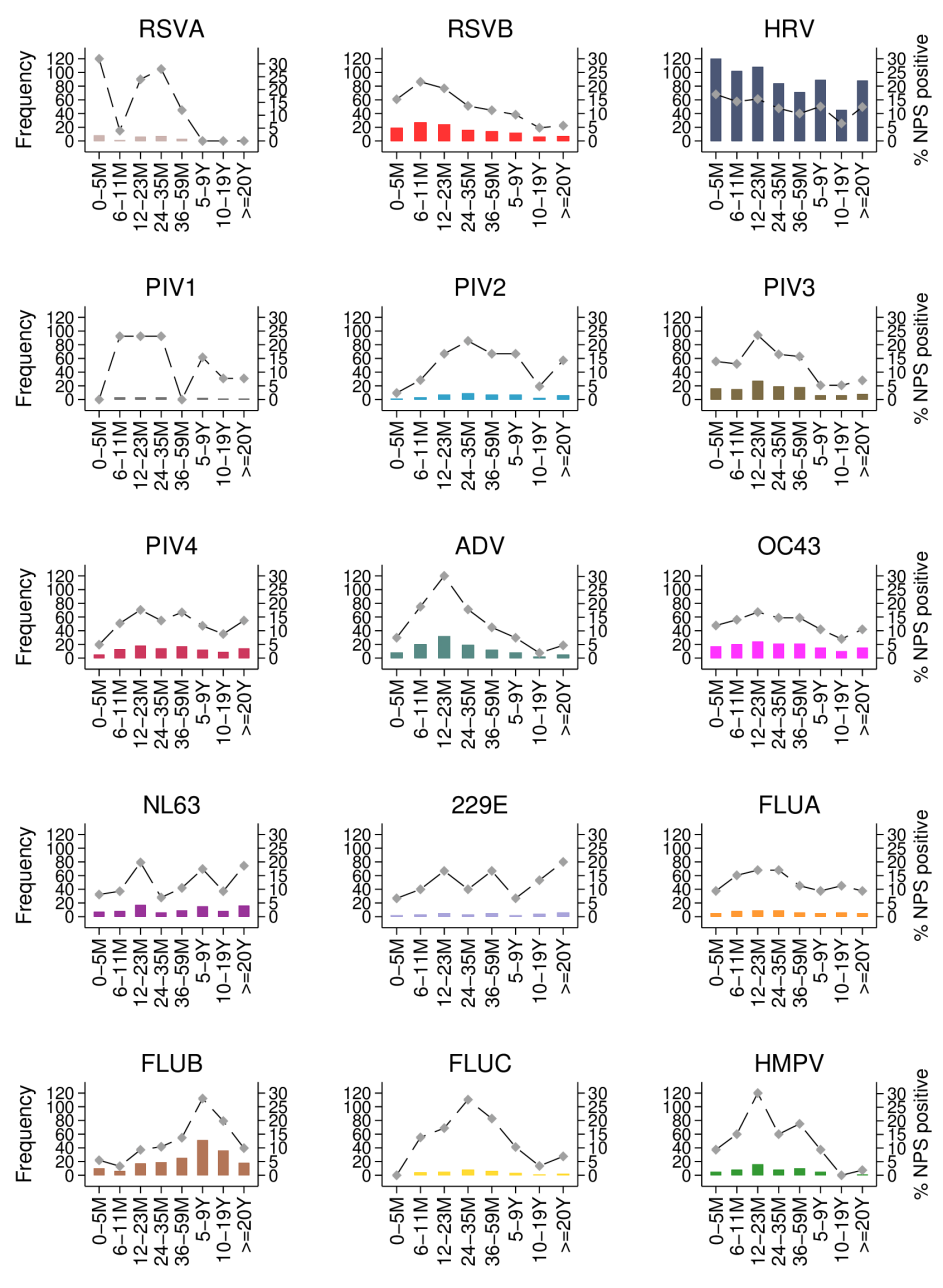
Age-distribution of detections of 15 virus targets in nasopharyngeal swabs (NPS) identified from ARI surveillance at nine health facilities in the Kilifi Health and Demographic Surveillance System, Kenya, January to December 2016. The primary Y axis shows frequency while secondary Y axis shows proportion in each age group. RSVA, RSV group A; RSVB. RSV group B; HRV, human rhinovirus; PIV1–PIV4, parainfluenza virus types 1–4; ADV, adenovirus; OC43, human coronavirus OC43; NL63, human coronavirus NL63; E229, human coronavirus E229; FLUA-FLUC, influenza viruses types A–C; HMPV, human metapneumovirus.

The age distribution of detections showed variation between viruses ( Figure 4). For example, RSV B was most frequent among participants 0 to 23 months, adenovirus in children 6 to 35 months, and influenza B in those aged 5 to 19 years.

Multiple viruses were detected in 4.4% of the participants (n=246), with 2, 3 and 4 co-infecting viruses detected in n=221, n=19 and n=6 of the participants, respectively. Rhinovirus occurred in 153 participants with multiple viruses; coronaviruses in 131 participants; adenovirus in 55 participants; parainfluenza virus type 3 in 36 participants and RSV B in 28 participants. Rhinoviruses and adenoviruses co-infected with all the other 14 target viruses while RSV group B was found not to co-infect with all, except HMPV.

Only 1.4% of the participants (n=33) with a virus-positive NPS sample were referred to the KCH for specialized management. Treatment given to participants with ARI symptoms who tested positive for any of the virus targets were: antibiotics 81.4% (n=1947), antihistamines (chlorphenamine maleate) 60.4% (n=1437) and paracetamol 86% (n=2045). The most common antibiotic drugs prescribed were amoxicillin (39.7%, n=945) and trimethoprim/sulfamethoxazole (35%, n=834). Amoxicillin/clavulanic acid (0.3%, n=8) and ciprofloxacin (0.7%, n=16) were less commonly prescribed.

### Seasonality of the detected viruses

**Figure 5.**
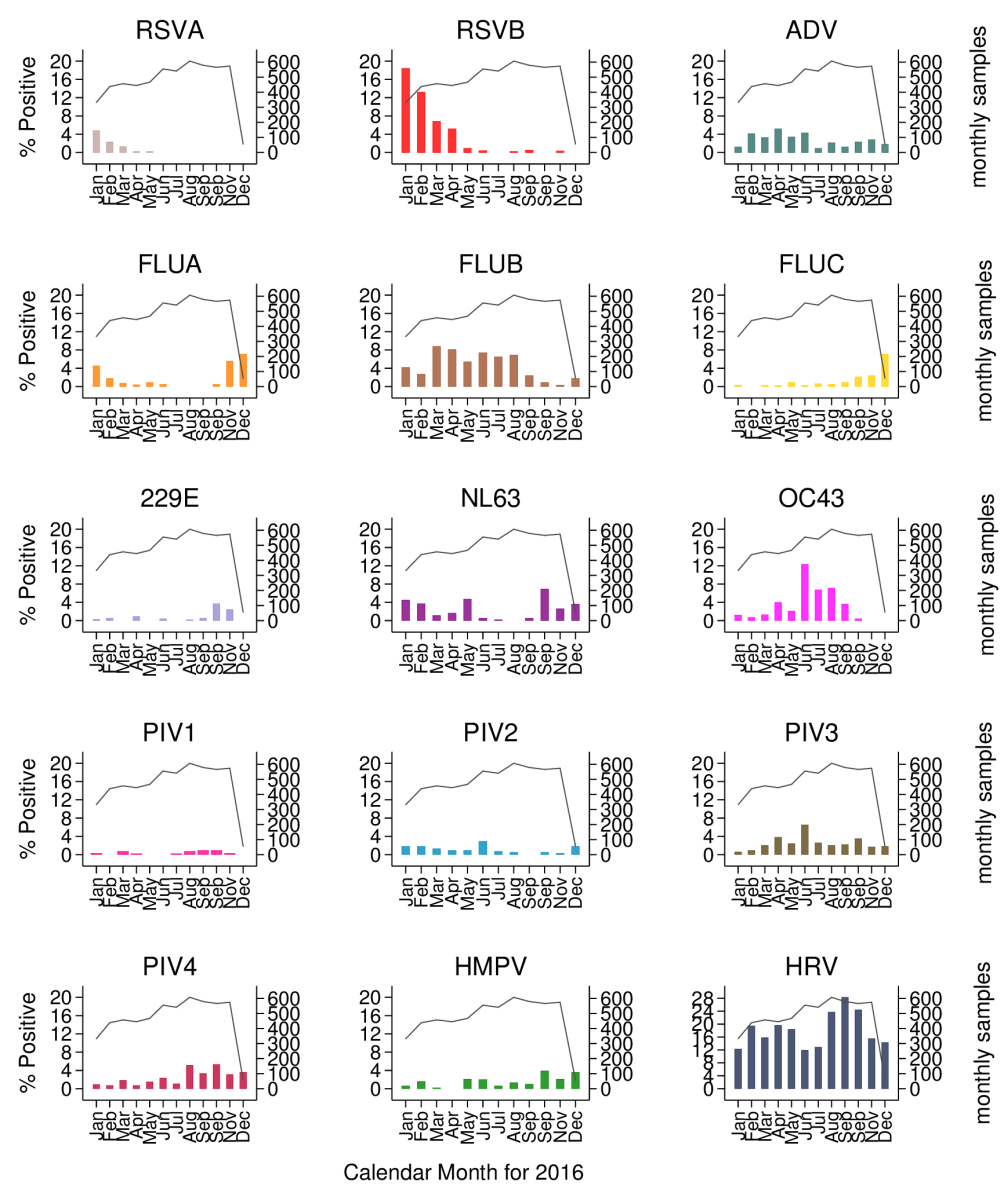
The distribution by month of the proportion of virus-positive nasopharyngeal swab samples over the period January to December 2016 for each of 15 virus targets, obtained through ARI surveillance at nine health facilities in the Kilifi Health and Demographic Surveillance System, Kenya. Secondary Y axis records number of samples collected from recruits per month. RSVA, RSV group A; RSVB. RSV group B; HRV, human rhinovirus; PIV1-PIV4, parainfluenza virus types 1-4; ADV, adenovirus; OC43, human coronavirus OC43; NL63, human coronavirus NL63; E229, human coronavirus E229; FLUA-FLUC, influenza viruses types A-C; HMPV, human metapneumovirus.

We observed a seasonal pattern in occurrence for some of the viruses ( Figure 5). Rhinovirus and adenovirus appeared to occur throughout the year. Influenza virus occurrence during the year differed between the types. Influenza type B, the most commonly detected influenza type, occurred predominantly between March and August. Influenza type C occurred mostly between September and December, while influenza type A was detected most commonly in January, February, November and December. RSV group B was predominant over RSV group A; cases for both groups arose predominantly in the first 4 months of the year. Coronavirus OC43 occurred mostly during the months of June to August and E229 at the end of the year; NL63 occurred least in the third quarter of the year. Seasonal patterns for parainfluenza viruses and HMPV were difficult to discern.

### Comparison of inpatient vs outpatient virus detections

**Figure 6.**
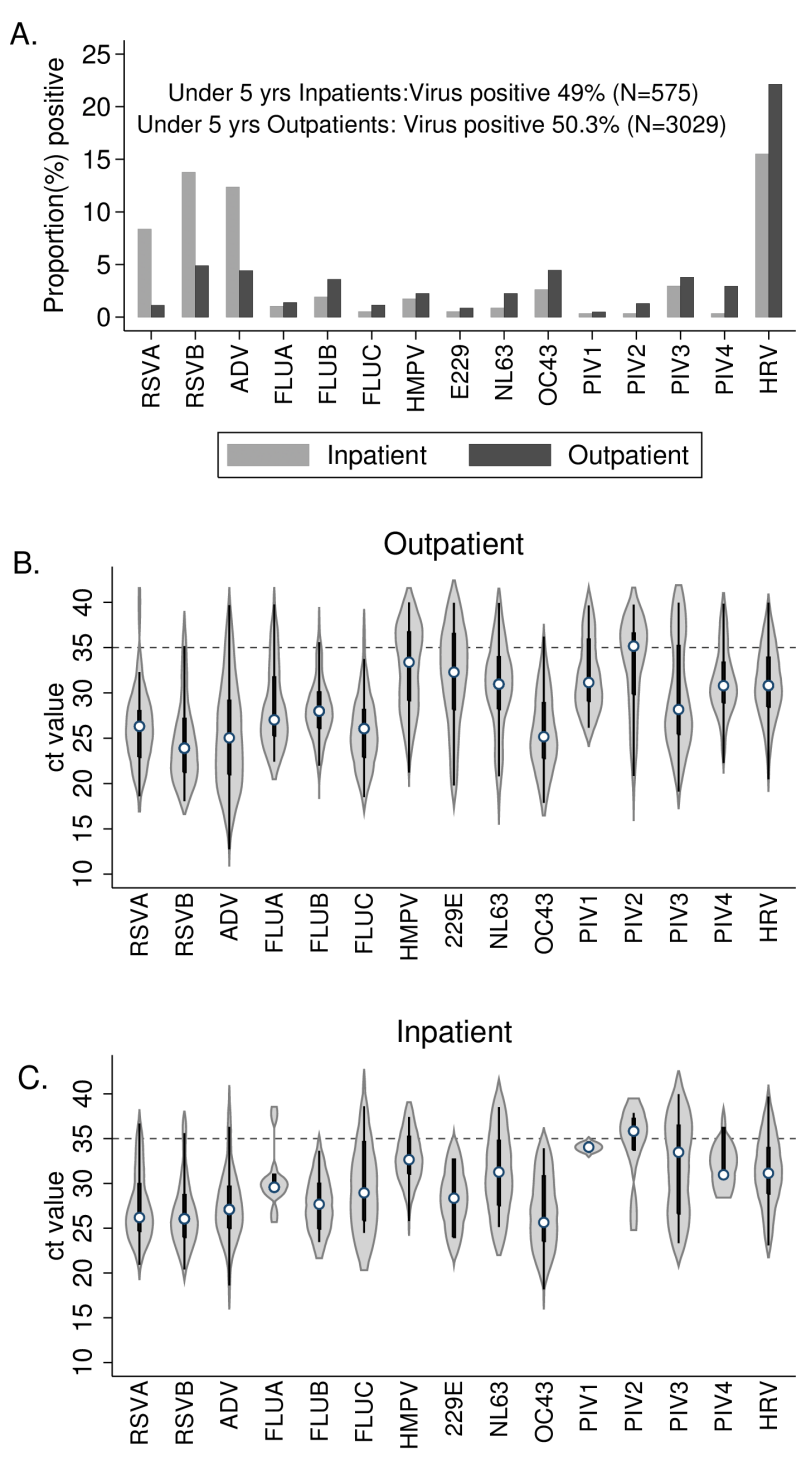
Virus surveillance comparison between inpatient and outpatient facilities for children under 5 years in the Kilifi Health and Demographic Surveillance System (KHDSS), Kenya, 2016. Panel A compares the proportion of nasopharyngeal swab samples positive for each of the 15 virus targets in samples collected from severe pneumonia admissions to Kilifi County Hospital (grey bars) and from outpatients presenting to nine health facilities (black bars). Violin plots show the distribution (median, IQR) for detectable rtPCR cycle threshold (Ct) values (i.e. Ct<=40) from respiratory samples for KHDSS outpatients (B) and for KCH inpatients (C). Threshold used for determining positive and negative samples shown by dashed line (Ct=35.0). RSVA, RSV group A; RSVB. RSV group B; HRV, human rhinovirus; PIV1-PIV4, parainfluenza virus types 1-4; ADV, adenovirus; OC43, human coronavirus OC43; NL63, human coronavirus NL63; E229, human coronavirus E229; FLUA-FLUC, influenza viruses types A-C; HMPV, human metapneumovirus.

A comparison of the distribution of viruses between the outpatient and the inpatient setting among children under 5 years of age is shown in Figure 6. A total of 49.0% NPS samples (n=282) from 575 inpatients with LRTI aged under 5 years were positive for one of the target respiratory viruses. RSV was the most common virus detected among hospitalized cases of ARI, with significantly higher prevalence 22.1% (n=127) than for outpatients 6.0% (182) (χ ^2^(1)=159.4, P=0.0001). Adenovirus was also of high prevalence and significantly more common 12.4% (n=71) than in outpatients 4.4 % (134) (χ ^2^(1) =56.6, P=0.0001). Rhinovirus was at high prevalence in both settings, but more so in the outpatients. Coronavirus OC43 and parainfluenza virus type 4 were more prevalent in outpatient than inpatients. All remaining viruses, though proportionally less common, showed higher prevalence in the outpatient setting. The distribution of Ct values for each virus target was similar for both inpatient and outpatient samples.

## Discussion

In this study on the coast of Kenya, we describe the frequency of occurrence, and the spatial and temporal distribution of 15 viruses associated with respiratory illness in the outpatient setting. We also compare the distribution of viruses associated with respiratory infection among children under 5 years of age in the outpatient and inpatient settings.

To provide baseline data for investigating the transmission patterns and pathways of spread for common respiratory viruses, we used molecular techniques to detect viruses circulating throughout a well-defined population of nearly 300,000 inhabitants in an area of ~900 km ^2^ in rural coastal Kenya. We observed a prevalence of 42% virus infection among the selected patients presenting with symptoms of ARI in outpatient departments of nine health facilities. These findings indicate that viruses are prominently associated with respiratory tract infections of sufficient severity for individuals to seek medical attention within this community.

Consistent with observations elsewhere ^2, 5, 24^, most of the ARI outpatients in this study were young children under 5 years; 50.3% of the participants in this age group had a virus positive NPS sample. This demonstrates that the under 5’s are a highly vulnerable age group for medically important ARI. The results are comparable to those from the outpatient health facilities in refugee camps and at a national referral hospital, in Kenya, where the proportions of virus-positive NPS samples in those under 5 years old with cases of ARI was 49.8% ^25^ and 54% ^26^, respectively. This age group appears to experience the highest burden of acute illnesses linked to the studied respiratory viruses and should therefore be a focus for future intervention strategies. However, additional data should be collected on the social and economic burden of these viruses, such as days of school missed, medical costs and time off work for caregivers.

Of all virus-positive patients presenting to the outpatient facilities, only a small proportion (30%) were in education (mostly kindergarten or primary.) This is of interest as the school age groups might be expected to be important agents in the community spread of infectious disease, certainly given their high rates of contact through which transmission is presumed to be effected ^27, 28^. There is need for empirical evidence to define the relationship between contact rates and respiratory virus transmission.

The most frequently detected virus in this study was rhinovirus. This is in accordance with previous community-based studies, for example the Tecumseh project in Michigan ^29^. Serological and molecular epidemiological studies show rhinoviruses to exist as many types (currently over 160) ^10, 21^, with little cross-type protection (amongst those classified immunologically), which may explain the high prevalence and absence of seasonality in respiratory illness caused by rhinovirus infection in the community. Persistence of rhinovirus might be ascribed to the frequent introduction of new virus strains into the community unconstrained by prior circulation of other types.

In this study, we did not see any major difference in distribution of viruses across the health facilities. This could have been attributed to the relatively small size of the demographic surveillance system area (891 km ^2^). This allows for the possibility of population mixing and frequent interactions, especially during social events, leading to the rapid spread of viruses across the KHDSS area. However, definitive understanding of the temporal and spatial patterns of spread requires the addition of sequence data to infer relatedness of circulating viruses.

Influenza, RSV and coronavirus exhibit a clear seasonality pattern in occurrence, whereas rhinovirus and adenovirus are detected throughout the year. Of note is that during the first quarter of the year, other than rhinovirus, RSV is predominant amongst the detected viruses. Currently little is known about the mechanisms underlying virus dominance, interaction, co-existence and competition. Studies are warranted to investigate occurrence and interactions of multiple respiratory viruses in the nasopharynx of the individual over time (i.e. across the seasons), and to explore the possible effect of eliminating a virus such as RSV through vaccination. Such information will be useful to guide policy on priority respiratory viruses to focus for intervention.

We also find the wide use of antibiotics to treat majority of patients presenting with symptoms of ARI most likely caused by viruses. This raises concern over antimicrobial stewardship, with increased risk of antimicrobial resistance to first-line antibiotic agents and unnecessary use of expensive second-line antibiotics in treating mild acute respiratory disease.

In contrast to the outpatient setting, where rhinovirus is the most common virus associated with ARI among children under 5 years; in the inpatient setting, RSV and adenovirus are the leading cause of severe respiratory illness. From the long-term in-patient surveillance at KCH the observation for RSV is not unusual, but 2016 had an unusually high occurrence of adenovirus cases (data not shown for other years). The pattern in distribution of virus load (equated to Ct values) suggests that the cut off of 35.0 for the MPX real time PCR diagnostic method is generally suitable for all viruses, both in outpatient and inpatients (i.e. irrespective of disease severity and possible viral load), excepting for HMPV, PIV-1 and CoV 22E in outpatients and PIV-2 in inpatients and outpatients, where sensitivity may be an issue and a contributor to low prevalence in this study.

The major limitation of this study is that data are for 1 year only and caution should be applied in inferring seasonal patterns. This is exacerbated by the low numbers of participants recruited in December due to countrywide industrial action by nurses. In addition, there is competition for viruses due to the design of sampling we used, of selected 15 samples per facility per week. An epidemic for one target virus might influence the proportions of other viruses observed but this might not necessarily imply seasonality. The detection of multiple viruses in one individual makes it difficult to determine the viral pathogen responsible for the respiratory illness at the time of recruitment. Only a sub-sample of all ARI presentations were recruited and the underlying denominator not recorded, which prevents an estimation of community incidence of presentations that would be useful for comparative purposes.

## Conclusions

In a sample of 5647 participants, about 40% of the ARI outpatient visits to the Kenyan Coast were associated with respiratory virus infection. Virus ARI is predominant among children <5 years, and relatively uncommon amongst school-going children. Rhinoviruses, influenza viruses, parainfluenza viruses, coronaviruses and RSV are most commonly associated with ARI over one year of community surveillance, whereas RSV and adenoviruses are the predominant respiratory virus among hospitalized patients with ARI. Virus occurrence (temporal and age-related) is similar across all facilities within the KHDSS area. Studies of the socio-economic implications of this burden are warranted, especially for rhinovirus infections that predominated. Further analysis of virus sequence data will delineate patterns of spread of viruses causing ARI illness in this setting.

## Data availability

The replication data and analysis scripts for this manuscript are available from the Harvard Dataverse: https://doi.org/10.7910/DVN/ZX7NS4 ^30^. As the dataset contains potentially identifying information on participants, it is stored under restricted access. Details on eligibility for access and a request form are available from http://kemri-wellcome.org/about-us/#ChildVerticalTab_15 for consideration by our Data Governance Committee ( dgc@kemri-wellcome.org).

## Supplementary material

**Supplementary File 1.** Biodata questionnaire used in this study.
