# Supplementary material for: Surveillance of respiratory viruses in the outpatient setting in rural coastal Kenya: baseline epidemiological observations
Source: Wellcome Open Res. 2018 Jul 25;3:89. [Version 1] doi: 10.12688/wellcomeopenres.14662.1 (PMC6081997; doi:10.12688/wellcomeopenres.14662.1)
Supplement: Supplementary file 1 [file wellcomeopenres-3-15964-s0000.tgz › 12dccaba-f373-4a9e-b463-8c2265ec97ac.docx]

**Health facility Clinical Assessment and Data collection form**

**Study lay Title: Investigating patterns of spread of viral respiratory** **infections in Kilifi County**

Date today (DD/MM/YYYY) __ __ /__ __/ 20__ __ Time: ___:___hrs

***Section I: Person details (****Field worker to fill****)***

Patient Names: 1____________________ 2___________________ 3___________________

Study Person id: __ __ __ __ ___ Date of birth: __ __ __ __ ___ __ Sex: ____ (M/F)

DSS PID: __ __ __ __ __ __ __ Residence (Village): _____________

HM Name: __ __ __ __ __ __ ____ ____ __ __ __ __ Location:_ _ _ __ __ __ ______

ID NO: __ __ __ __ __ __ ____ ____ __ __ __ __

Facility Name: __ __ __ __ __ __ ___ Facility Code: __ __ __ __

If Child;

Mothers Name: __ __ __ __ __ __ ____ __ __ __ __ __ __ __ ____ __

For Non-DSS Residents;

Specify County: ___________________ Residence (Location): ___________________

Recent travel outside your location in last 2 weeks: [ ] Y/N

If yes, specify where from: ___________________

Are you in school?: [ ] Y/N

If yes, specify education level

Education level: 1. Kindergarten; 2. Primary; 3. Secondary; 4. Tertiary

5. None

If Primary, Specify: 1. Name of School: __________________ 2. Class: _________________

3. Location of School: ___________________

Are there other persons in your household who have symptoms of ARI? [ ] Y/N

If yes, are they in school? [ ] Y/N

If yes, specify the number in each education level

Education level: 1. Kindergarten 2. Primary 3. Secondary 4. Tertiary

If Primary/Kindergarten, Specify:

1. Name of School: (i) ____________ (ii)____________________

(iii)_________________________ (iv) ___________________________

2. Class: (i) _______________________ (ii)_________________________

(iii) ___________________________ iv) _________________________

3. Location of School: (i)_________________________ (ii)___________________ (iii) _____________ ( iv) _______________________________

Ethnicity (specify): ___________________

Occupation (Specify): ___________________

Brought to health facility by (caretaker names): _______________________________

Relation to the patient: ________________

Referred by: ____________FW/Self/Study clinician

***Section II: Anthropometrics*** (*Field worker to fill****)***

1. For children <12 years

Weight [ ] [ ] [ ]**.** [ ] [ ] *kg*

MUAC [ ] [ ]**.** [ ] *cm*

Head Circumference [ ] [ ]**.** [ ] *cm*

Height/Length [ ] [ ] [ ]**.** [ ] *cm*

1. Oxygen saturation [ ] [ ] [ ] %
2. Heart rate [ ] [ ] [ ]/min
3. Axillary temperature [ ] [ ]**.**[ ] ^0^C
4. Respiratory rate [ ] [ ]/min

***Section III: Clinical assessment*** (Clinician to fill)

1. How long has the patient been sick? [ ] [ ] days
2. What are the main complaints? (*Please indicate Yes or No*)

Fever [ ] y/n

Cough [ ] y/n

Nasal discharge/congestion [ ] y/n

Difficulty in breathing [ ] y/n

Nasal flaring [ ] y/n

Sore throat [ ] y/n

Chest wall in drawing [ ] y/n

Crackles [ ] y/n

Wheezes [ ] y/n

Unable to feed [ ] y/n

Head nodding [ ] y/n

Cyanosis [ ] y/n

Unable to talk in complete sentences [ ] y/n

1. Conscious level (*please tick one)*

Alert Lethargic Prostrate Unconscious

1. Other complaints *(specify in the box below)*
2. Laboratory tests:

Malaria test: *Indicate results as either positive, negative, equivocal or not done*

Rapid diagnostic test: [ ]

Peripheral blood slide: [ ]

1. Primary diagnosis (s*pecify*): ­­­­­­­­­­­­­ _____________________________________________
2. Other diagnoses *(specify in the box below)*
3. Treatment given *( List all the drugs prescribed in the box below)*
4. Does the person require referral for hospital(KDH) admission [ ] (y/n)
5. Initials of the clinician reviewing the patient [ ]

**Section IV: Specimen Collection** (*Field worker to fill****)***

1. Is the primary diagnosis ARI? [ ] (y/n)
2. If yes, has the swab been collected: [ ] (y/n)
3. If nasal sample is not collected specify reason: ______________________________
4. Nasal specimens collected by *(indicate your initials*) [ ]

**Notes for the completion of the Health facility study Clinic visit proforma**

**General instructions**

- This clinical assessment form is to be filled for all clinic visits made by the HF study participants presenting at the following health facilities, Matsangoni, Ngerenya, Sokoke, Mtondia, Mavueni, Jaribuni, Chasimba and Junju with symptoms of acute respiratory infection.
- The form will be filled by the study field worker assisted by the clinician.
- Nasal or oral specimen should be taken to all eligible participants with ARI symptoms.

**Section I: Patient details**

Indicate the patient ***full names***, the ***person id*** (unique study number) and the ***DSS PID***. Using the DSS PID search and match, then upload participant demographic details from the KHDSS census database.

***Sex:*** Indicate whether male or female

**Date of birth:** Indicate the day month and year of birth.

***Residence:*** This should be the village of residence

***Caretaker:*** Refers to the person who brought or accompanied the patient to the clinic. Note the ***relation*** of the caretaker with the patient. He/she could be from the same household as the patient or not.

***Referral***: Establish who referred the patient to the clinic. Whether it’s the study FWs, clinician or it’s a self-referral

***Section II: Clinical assessment***

***Weight:*** measured in Kgs into 2 decimal places. Ensure the weighing scales are calibrated every week.

***MUAC:*** mid-upper arm circumference; reported in cm. This will only be collected in children who are below 13 years old.

***Height/Length:*** measure in cms

***Oxygen saturation:*** Use pulse oximeter to measure oxygen saturation. Recheck if the oxygen saturation is below 95% and if it is less than 50% there is likely to be a problem with the Oximeter – repeat measurement. If less than 90% repeat the procedure and compare the two readings. Ensure person is at rest and at ease.

***Heart rate****: or pulse rate:*  Use the readings from the pulse oximeter or count peripheral pulse rate for one complete minute!

***Axillary temperature:*** Take axillary temperature using a digital thermometer e.g. accuracy 37.3

***Respiratory rate:*** Also referred to as breaths per minute. Count respirations for one complete minute. Ensure person is in calm stated and rested before starting. If a child it should not be agitated or crying for example. Record the breathing rate as accurately as possible. Ensure the participant has settled before recording. Assess if child has fast breathing (i.e. >50 for all ages, and >40 if 12 months or over).

***Duration sick:*** Enquire the duration (number in days) the patient has been ill. If the person has been ill for more than a month exclude from the study.

***Main complaints:*** during the clinical assessment of the illness establish whether any of the listed respiratory-related signs and symptoms are reported.

***Other complaints:*** Note the presence of any other complaints which might be accompany respiratory illness e.g. diarrhoea, vomiting

**Laboratory tests:** Please indicate any malaria parasite test results for ***Rapid diagnosis*** and or ***blood slide*** as either positive, negative, equivocal or not done.

***Primary diagnosis:*** Indicate the final/main diagnosis based on patient complains and any laboratory tests done.

***Other diagnoses:*** Note any other diagnosis where applicable.

***Referral:*** Indicate if the person requires further clinical care (hospital admission) in the Kilifi County Hospital.

**Section III: Specimen Collection**

Collect a nasal and oral specimen if the person has symptoms of ARI. If nasal sample is not collected specify ***reasons***. Please indicate the **initials** of the FW/clinician collecting the sample.
