# Supplementary material for: Surveillance of respiratory viruses in the outpatient setting in rural coastal Kenya: baseline epidemiological observations
Source: Wellcome Open Res. 2018 Jul 25;3:89. [Version 1] doi: 10.12688/wellcomeopenres.14662.1 (PMC6081997; doi:10.12688/wellcomeopenres.14662.1)
Supplement: Peer review history

# Peer review: Referee response for version 1

**DOI:** 10.21956/wellcomeopenres.15964.r33628

**Published:** 2018-08-06

**Authors:** David P. Moore

**Affiliations:** 1 Medical Research Council, Respiratory and Meningeal Pathogens Research Unit, University of the Witwatersrand, Johannesburg, South Africa; 2 Department of Paediatrics, Chris Hani Baragwath Academic Hospital and University of the Witwatersrand, Johannesburg, South Africa

This study on the molecular epidemiology of respiratory viral illness in community and hospital settings in costal rural Kenya is an important addition to the growing literature on respiratory viral epidemiology in sub-Saharan African children. Future publications from this group of researchers is eagerly anticipated, particularly with regards tracking temporality and seasonality of viral prevalence over multiple years, and detailing the frequently-occurring viral-viral co-detections in this cohort.

 Kenya is generally known to be a low human immunodeficiency virus type 1 (HIV) prevalence setting. It would, however, be useful to factor in HIV status of study participants in future studies from this well-delineated catchment area.

 Point for clarification in the analysis:

 1. In the first sentence of the **Results **section, 5647 participants were enrolled – considerably fewer than the 7000 participants sought in the sample size calculation: how this ‘under-count’ impacted on the ability to establish certainty of statistical inference needs to be mentioned in the Discussion section;

 2. **Figures 6 B** and **6 C** seem superfluous, as there is no text which describes the statistical comparison between pathogen density and inpatient or outpatient status. Suggest add in one or two sentences regarding this analysis in the last paragraph of your **Results **section.

 Recommended minor stylistic adjustments:

- In the first sentence of the **Introduction**, rather state ‘and with the majority of the burden’;
- In the second sentence of the **Introduction**, rather state ‘are recognized as the major cause’;
- In the first sentence of the second paragraph of the **Introduction**, rather state ‘Given this context, it is likely that greater emphasis will be placed on the control of virus-associated ARI.’;

3. In order to strengthen the important assertions made in sentence four of the second paragraph of the **Introduction**, especially with regards ‘social distancing’, I would suggest that the authors reference this sentence;

 4. In the last sentence of paragraph three of the **Introduction**, suggest reword as ‘Here we present the baseline results of 1 year of respiratory virus epidemiological surveillance at health facilities within a well-defined coastal Kenyan population, and compare them with contemporaneous data from inpatient admissions to Kilifi County Hospital (KCH).’;

 5. In the fourth sentence of the first paragraph of **Patient recruitment and specimen collection**, reword as ‘they awaited review’;

 6. In the last sentence of the second paragraph of **Patient recruitment and specimen collection**, I would suggest that the authors reference their proportional split between respiratory viruses;

 7. In the second sentence of the paragraph on **Statistical analysis**, suggest reword as ‘proportion of virus positives’;

 8. In the third sentence of the paragraph on **Statistical analysis**, insert a space between the preceding full stop and the words ‘Chi-squared’;

 9. In **Table 1**, it makes logical sense to reorder the columns according to order in which a clinician would examine a patient, starting with history and moving to physical examination: I propose that an optimised order would be:  ‘Cough’, ‘Difficulty breathing’, ‘Fever’, ‘Nasal discharge’, ‘Nasal flare’, ‘Chest indrawing’, ‘Crackles’, ‘Wheeze’;

 10. On **page 6**, in the second last sentence of column one (starting ‘The number of participants in each facility…’ gives a *Χ* ^2^ degree of freedom of 11: is this in fact correct? My reading is that this should be 8 (in view of 9 included outpatient facilities having been included in the sampling strategy). Please correct if appropriate;

 11. Also, close the gap between ‘ *Χ* ^2^(8/11)’ and ‘ =78.26’;

 12. In the first sentence under **Virus detection**, reword as ‘respiratory virus targets’;

 13. In **Table 2**, add a space between ‘positive’ and ‘(n)’ in the second cell in the top row, and between ‘Total’ and ‘(n)’ in the sixth cell. Widen the Table slightly to accommodate a slightly wider columns;

 14. In the first line on **page 7**, suggest close the gaps between ‘ *Χ* ^2^’, ‘ (1)’, and ‘ =177.3’;

 15. On **page 7**, the last sentence of the first paragraph (which describes the pilot phase of the study in December 2015) does not seem to add to the ‘meat’ of the results: suggest delete this sentence;

 16. In the last sentence of the fourth paragraph on **page 7**, suggest reword to ‘except with HMPV.’;

 17. In the second sentence of the second column on **page 7**, suggest reword influenza A activity to ‘while influenza A had biphasic activity in January/February and November/December’;

 18. In the first sentence under the heading **Comparison of inpatient vs outpatient virus detections**, suggest reword to ‘inpatient settings’;

 19. In the fourth sentence under the heading **Comparison of inpatient vs outpatient virus detections**, suggest close the gap between ‘ *Χ* ^2^(1)’ and ‘ =56.6’;

 20. In the last line on **page 7**, suggest qualify as ‘, both in Kenya,’;

 21. In the legend to **Figure 2**, replace the full stop after ‘RSVB’ with a comma and change ‘E229’ to ‘229E’;

 22. In the legend to **Figure 3**, replace the lower case ‘e’ in ‘229e’ with an upper case ‘E’;

 23. In the first line on **page 8**, suggest reword as ‘under 5 years old with ARI were 49.8% and 54%, respectively.’;

 24. In the first sentence of the second paragraph on **page 8**, place the full stop outside the closing parentheses: ‘kindergarten or primary).’;

 25. In the first sentence of the third paragraph on **page 8**, suggest change wording from ‘in accordance’ to ‘in keeping’;

 26. In the legend to **Figure 4**, replace the full stop after ‘RSVB’ with a comma and change ‘E229’ to ‘229E’;

 27. In **Figure 5**, the same seasonal (grey) line plot is included in each of the graphs and represents the total number of samples collected per month in the study: this should be mentioned in the Figure legend;

 28. In legend to **Figure 5**, replace the full stop after ‘RSVB’ with a comma and change ‘E229’ to ‘229E’;

 29. In **Figure 6 A**, suggest quote the percentage ‘49%’ to one decimal place, and add a space between ‘Inpatients:’ and ‘Virus’ in the text superimposed on the plot area;

 30. In the legend to **Figure 6**, replace the full stop after ‘RSVB’ with a comma and change ‘E229’ to ‘229E’;

 31. In the first sentence of the second paragraph on **page 13**, suggest reword as ‘We observed a high prevalence of antibiotic prescribing to outpatients presenting with ARI most likely cause by viruses.’;

 32. In the second sentence of the third paragraph in **page 13**, suggest reword to ‘Based on long-term in-patient surveillance’;

 33. In the third sentence of the third paragraph in **page 13**, suggest replace ‘MPX’ with ‘multiplex’, as the abbreviation ‘MPX’ has not been defined before in the text;

 34. In the third sentence of the third paragraph in **page 13**, suggest reword to ‘both in outpatients and inpatients’;

 35. In the third sentence of the third paragraph in **page 13**, correct ‘CoV 22E’ to ‘CoV 229E’;

 36. In the second sentence of the second column on page 13, suggest reword as ‘respiratory virus among children hospitalized with LRTI’.

I have read this submission. I believe that I have an appropriate level of expertise to confirm that it is of an acceptable scientific standard.

# Peer review: Referee response for version 1

**DOI:** 10.21956/wellcomeopenres.15964.r33577

**Published:** 2018-07-31

**Authors:** Ting Shi

**Affiliations:** 1 University of Edinburgh, Edinburgh, UK

This is a well described manuscript reporting the epidemiological data of respiratory viruses at nine outpatient health facilities in rural coastal Kenya. More than half (53.7%) of participants with ARI symptoms were from children younger than 5 years. The most common respiratory viruses detected were rhinovirus, influenza virus, coronavirus, parainfluenza virus, respiratory syncytial virus (RSV) and adenovirus. In comparison group with young children admitted to hospitals, the frequency of RSV and adenovirus was significantly higher. However, a few points need to be clarified.

1. Would be nice to know the severity of participants with multiple viruses and see whether there is an association. What about the multiple viruses infection in inpatients?
2. Newborns aged less than 7 days were excluded. Would this affect the detection rate of respiratory viruses in infants? Would this underestimate the burden?

The detection of viruses in participants with ARI symptoms does not necessarily indicate a causal attribution of these viruses (association ≠ causality). Would be nice to add a few sentences discussing this point.

I have read this submission. I believe that I have an appropriate level of expertise to confirm that it is of an acceptable scientific standard.
